# Development and Mechanical Characterization of a Jute Fiber-Reinforced Polyester Composite Helmet Produced by Vacuum Infusion

**DOI:** 10.3390/polym18020235

**Published:** 2026-01-16

**Authors:** Robson Luis Baleeiro Cardoso, Maurício Maia Ribeiro, Douglas Santos Silva, Raí Felipe Pereira Junio, Elza Monteiro Leão Filha, Sergio Neves Monteiro, Jean da Silva Rodrigues

**Affiliations:** 1 Materials Engineering Program, Federal Institute of Education, Science and Technology of Pará—IFPA, Avenida Almirante Barroso, 1155, Marco, Belém CEP 66093-020, PA, Brazil; rlbaleeiro@gmail.com (R.L.B.C.); elza.filha@ifpa.edu.br (E.M.L.F.); jean.rodrigues@ifpa.edu.br (J.d.S.R.); 2 Federal Institute of Education, Science and Technology of Pará—IFPA, Estrada do Icuí Guajará, Ananindeua CEP 67125-000, PA, Brazil; mauricio.maia@ifpa.edu.br; 3 Department of Materials Science, Military Institute of Engineering—IME, Praça General Tibúrcio, 80, Praia Vermelha, Urca, Rio de Janeiro CEP 22290-270, RJ, Brazil; raivsjfelipe@ime.eb.br (R.F.P.J.); sergio.neves@ime.eb.br (S.N.M.)

**Keywords:** polyester composite, jute fiber, vacuum infusion, mechanical properties, sustainable materials

## Abstract

This study presents the development and mechanical characterization of a full-scale helmet manufactured from a polyester matrix composite reinforced with woven jute fabric using vacuum infusion. Laminates with two and four reinforcement layers were produced and assembled using four joining configurations: seamless, stitched, bonded, and hybrid (bonded + stitched). Tensile tests were performed according to ASTM D3039, while frontal and lateral compression tests followed ABNT NBR 7471, aiming to evaluate the influence of laminate thickness and joining strategy on mechanical performance. In tension, the seamless configuration reached maximum loads of 0.80 kN (two layers) and 1.60 kN (four layers), while the hybrid configuration achieved 0.79 kN and 1.43 kN, respectively. Stitched and bonded joints showed lower strength. Under compression, increasing the laminate thickness from two to four layers reduced frontal elongation from 15.09 mm to 9.97 mm and lateral elongation from 13.73 mm to 7.24 mm, corresponding to stiffness gains of 50.3% and 87.3%, respectively. Statistical analysis (ANOVA/Tukey, α = 0.05) confirmed significant effects of thickness and joint configuration. Although vacuum infusion is a well-established process, the novelty of this work lies in its application to a full-scale natural-fiber helmet, combined with a systematic evaluation of joining strategies and a direct correlation between standardized tensile behavior and structural compression performance. The four-layer hybrid laminate exhibited the best balance between strength, stiffness, and deformation capacity.

## 1. Introduction

The search for sustainable and economically competitive structural materials has driven increasing interest in polymer composites reinforced with natural fibers. These materials, commonly referred to as natural fiber-reinforced polymer composites (NFRPCs), offer advantages such as low density, renewability, reduced environmental impact, and favorable specific mechanical properties. Among lignocellulosic reinforcements, jute stands out due to its wide availability, low cost, high tensile strength, and capacity to improve stiffness and energy absorption in polymer matrices [1,2,3,4]. As a result, jute-reinforced polyester and epoxy composites have been increasingly investigated for applications in the automotive, civil construction, sporting goods, and lightweight structural sectors [5,6].

It is important to clarify that, although jute fibers are biodegradable by nature, the biodegradation process of jute fiber-reinforced polymer composites occur predominantly under specific environmental conditions, such as prolonged exposure to moisture, microorganisms, ultraviolet radiation, and elevated temperatures. Several studies have demonstrated that, when embedded in a thermoset polymer matrix such as polyester or epoxy, the biodegradation of jute fibers is significantly delayed due to the protective effect of the matrix, which limits water diffusion and microbial activity. As a result, the mechanical properties of jute-based composites remain relatively stable over short- and medium-term service periods, particularly under indoor or controlled-use conditions typical of structural and protective applications. Therefore, the biodegradability of jute fibers should be regarded as a long-term environmental advantage rather than a factor that compromises the immediate mechanical integrity of the composite structures evaluated in this study.

Despite these advantages, the processing of natural-fiber composites presents well-known challenges, particularly regarding fiber impregnation, void formation, thickness control, and interlaminar variability. Many studies reported in the literature rely on hand lay-up techniques, which are prone to uneven resin distribution, poor consolidation, and limited reproducibility, especially when applied to curved or three-dimensional components [7,8,9]. Such limitations hinder the adoption of natural fibers in structurally demanding applications, where consistency and reliability are critical.

Vacuum-assisted manufacturing techniques, such as vacuum infusion, have been widely adopted to overcome these issues by improving resin flow control, laminate consolidation, and dimensional accuracy. In recent years, vacuum infusion and related processes have been successfully applied to jute-based composites, including hybrid systems, nanomodified matrices, and advanced curing routes [10,11,12,13,14,15]. However, the majority of these investigations remain focused on flat laminates or mechanically simple geometries, with limited relevance to complex structural components.

Curved and closed structures, such as helmet shells, require highly controlled resin flow, reduced porosity, and proper consolidation to ensure adequate performance under tensile, compressive, and impact loading. Open literature consistently reports that porosity plays a critical role in governing the mechanical performance of natural-fiber-reinforced polymer composites. Increased void content has been shown to significantly reduce tensile strength, compressive stiffness, interlaminar shear strength, and impact resistance, primarily due to stress concentration effects, reduced effective load-bearing area, and premature crack initiation. Studies on jute- and flax-reinforced polyester and epoxy composites indicate that void contents above approximately 3–5% can lead to strength reductions ranging from 10% to more than 30%, depending on the loading mode and laminate architecture [6,7]. In curved and closed structures, uncontrolled resin flow and insufficient consolidation may locally increase porosity, further amplifying mechanical property degradation. Therefore, manufacturing routes that promote controlled resin impregnation and reduced void content—such as vacuum infusion—are essential to ensure reliable mechanical performance in natural-fiber composite structures.

In parallel, significant advances have been reported in composite joining technologies, particularly those involving consolidation and curing strategies under vacuum-assisted processes. Sam-Daliri et al. [16] demonstrated that co-curing techniques applied to unidirectional glass fiber-reinforced epoxy composite joints result in superior mechanical performance and damage tolerance compared to conventional adhesive bonding, due to enhanced interfacial continuity and reduced stress concentrations. Similarly, Chen et al. [17] investigated co-curing approaches without adhesive films for ultra-thin steel–CFRP hybrid joints, showing that surface modification combined with vacuum-assisted consolidation plays a decisive role in interfacial strength, load transfer efficiency, and joint durability. These studies highlight that joint performance in composite structures is strongly governed by curing strategy, interface quality, and consolidation conditions during manufacturing.

Within the field of personal protective equipment, this gap is even more pronounced. Studies involving natural-fiber composites rarely address helmet-like structures and, when they do, typically rely on manual lamination techniques without strict adherence to standardized mechanical testing protocols such as ASTM D3039 [18] or ABNT NBR 7471 [19]. Moreover, the mechanical integrity of joining strategies—such as stitching, bonding, or hybrid solutions—has received little attention, despite being a critical factor in closed-shell structures manufactured from natural fibers. Equally scarce are studies that establish a direct correlation between coupon-level mechanical properties and the structural response of full-scale protective components.

Although recent works have advanced the understanding of jute-based composites through multiscale modeling, hybridization strategies, surface modification, and performance prediction [20,21], the transition from material-level characterization to structural validation remains an open research challenge. In particular, the application of standardized tensile testing to predict the mechanical behavior of full-scale natural-fiber composite helmets has not been systematically explored.

It is important to emphasize that vacuum infusion itself is not proposed here as a novel manufacturing technique, as it is already well established for the production of polymer composite laminates. The originality of the present study lies in the application of vacuum-infused natural jute fiber composites to the manufacture of a full-scale helmet structure, combined with a systematic investigation of different joining configurations and their influence on both tensile behavior and structural compression performance under helmet certification standards. To the authors’ knowledge, no previous study has integrated standardized tensile testing with full-scale helmet compression assessment for natural-fiber-reinforced composite shells.

In light of these gaps, this study investigates the mechanical performance of a polyester/jute composite helmet produced by vacuum infusion, considering two laminate thicknesses (two and four layers) and four joining configurations: seamless, stitched, bonded, and hybrid (bonded + stitched). Tensile tests were conducted according to ASTM D3039 [18], while frontal and lateral compression tests followed the procedures established by ABNT NBR 7471 [19]. By correlating coupon-level tensile results with the structural response of the helmet, this work provides new insights into the feasibility, limitations, and optimization of natural-fiber composites for protective structural applications.

## 2. Materials and Methods

### 2.1. Materials

The fabrication of the prototype mold employed construction-grade gypsum, unsaturated polyester resin, and natural jute fiber as the primary constituent materials. The gypsum used is a widely applied binder in civil construction, particularly for precast components and drywall systems, due to its ability to provide a smooth surface finish and short setting time, typically ranging from 15 to 20 min. The material was procured commercially in 20 kg bags (Massafra Materiais de Construção, Belém, PA, Brazil). For mold preparation, a proportion of 14 kg of gypsum to 20 L of water was adopted, resulting in a mixture with adequate consistency for mold filling and dimensional stability during curing.

The resin used for the infusion process was an unsaturated polyester system, pre-accelerated and of medium reactivity, supplied by Center Glass Resinas e Fibras de Vidro (Belém, PA, Brazil). The resin is supplied in liquid form, exhibits low viscosity, and develops a brown coloration after curing. It presents high wettability in mats, rovings, and woven glass fiber fabrics, as well as excellent flow characteristics suitable for hand lay-up and spray-up applications, ensuring uniform fiber impregnation. According to the manufacturer, the resin has a Brookfield viscosity of 250–350 cP at 25 °C, a gel time of 10–15 min when catalyzed with 1% MEK-P, and an exothermic peak temperature of approximately 190 °C. Once cured, it forms high-strength composite materials and can incorporate mineral fillers, such as aluminum trihydrate (up to 30% to enhance fire resistance) or calcium carbonate (to reduce production cost).

Natural jute fiber was used as the reinforcing phase and acquired from the local market in Belém, PA, Brazil, processed by Indústria de Tecidos Castanhal, a well-established Brazilian manufacturer. The fibers were supplied in the form of a plain-woven fabric with 90° fiber orientation, without prior chemical treatment. The jute fabric exhibits a density of 1.308 g/cm^3^ and an average tensile load capacity of 40.80 N per yarn, demonstrating adequate mechanical characteristics for use in composite reinforcement. In addition to its mechanical performance, jute represents a sustainable alternative due to its renewable origin and reduced environmental impact compared to synthetic reinforcements.

### 2.2. Methods

#### 2.2.1. Mold Fabrication Procedure

The mold used for producing the prototype helmet was developed from an existing commercial motorcycle helmet. Due to the complex geometry of the component and its requirement for a smooth external surface, plaster molding was selected. This technique is widely adopted in civil construction and offers advantages such as ease of handling, dimensional stability after curing, and low acquisition cost, making it suitable for producing molds with good surface quality.

Initially, the objective was to manufacture a single-piece mold that would enable complete infusion of the prototype in a single step, thereby avoiding joint lines in the final composite structure. However, this approach would significantly increase the complexity of the infusion system and compromise the demolding process, especially given the undercut regions and curved surfaces inherent to helmet geometry. To ensure proper fabrication, infusion feasibility, and safe demolding, the molding strategy was adjusted, and the prototype was produced in two separate halves using an open-mold process.

**Figure 1:**
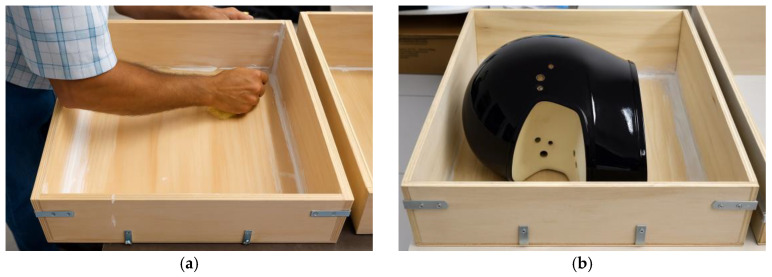
(**a**) Applying putty to the edges of the box, (**b**) Checking the space for molding the helmet.

For the preparation of the plaster mold, a containment structure was first constructed to hold the plaster mixture during casting. A low-cost plywood panel, readily available in the local market, was used to assemble the mold box. During assembly, small gaps were observed between the plywood joints. To prevent leakage and ensure proper mold integrity, wood adhesive was applied along the internal joints, followed by a sealing layer of commercial spackling compound, commonly used in civil finishing applications to block air passage and improve surface sealing prior to pouring the plaster mixture, as shown in Figure 1.

**Figure 2:**
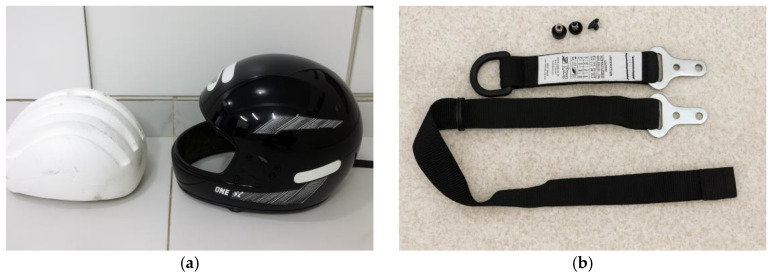
Safety equipment: (**a**) Styrofoam protection, (**b**) Chin straps.

In the commercial helmet selected as the reference model, the mounting holes for the chin strap and the visor were sealed to prevent the formation of undesired cavities in the mold. Additionally, all external adhesive labels were removed to avoid interference during the demolding process and to ensure an accurate reproduction of the original geometry. Figure 2 shows the safety devices.

**Figure 3:**
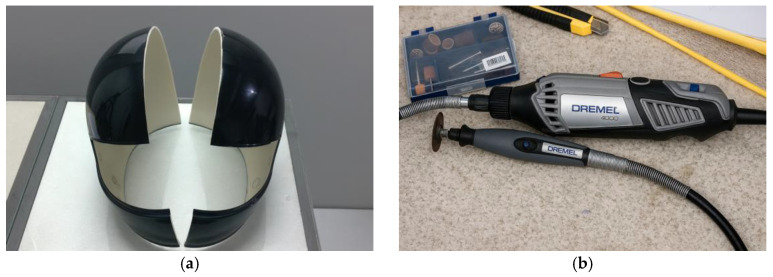
(**a**) Two-piece helmet, (**b**) Micro grinder with cutting disc.

The helmet shell was sectioned along its longitudinal axis using a rotary micro-grinder equipped with a cutting disc, as illustrated in Figure 3.

**Figure 4:**
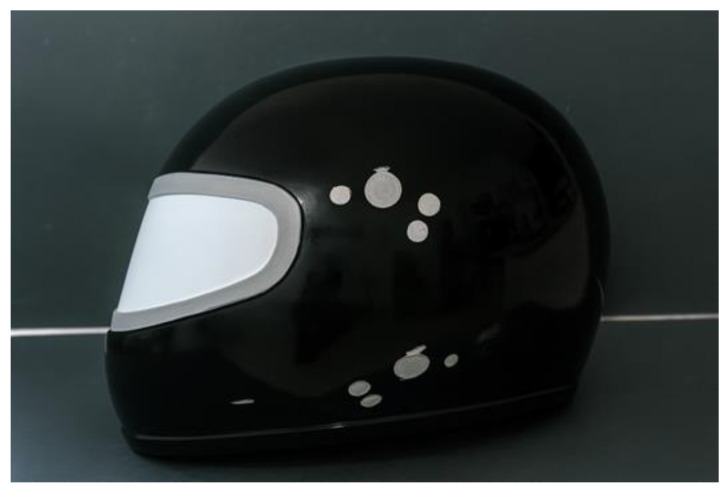
Helmet with sealed holes on the left and right sides for the visor opening.

After sectioning the commercial helmet, the openings in the shell were sealed, as shown in Figure 4, in order to obtain a smooth and continuous surface without protrusions or recessed areas on the mold.

**Figure 5:**
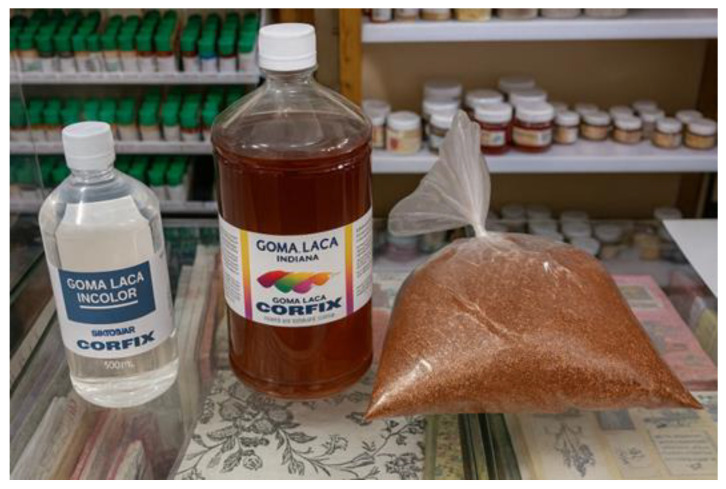
Shellac, release material for plaster.

After cutting the helmet model and sealing the visor area, the prototype molding process was initiated. Once the mold geometry was properly shaped, a release agent was applied to the model surface to facilitate demolding after the curing stage. For this purpose, shellac was employed—a natural release agent derived from the secretion of Indian lac insects (*Kerria lacca*). This material is readily available in the local market of Belém, PA, Brazil, in different forms: as a clear liquid, as a liquid containing small residues of insect wing fragments, and as granules, the latter being commonly used in traditional handicrafts. Figure 5 shows the different physical forms of shellac used in the process.

**Figure 6:**
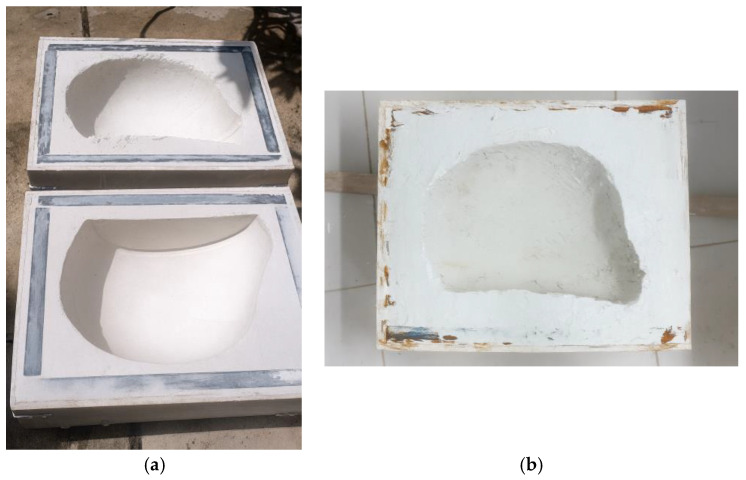
(**a**) Drying the plaster in the sun, (**b**) Controlled drying at 40° for 72 h.

For the composite production process using the vacuum infusion technique, the surface of the mold was carefully prepared through the application of release agents in different forms. Initially, a liquid mold release wax was applied over the entire surface, following a procedure of five successive coatings, each separated by drying and polishing intervals to ensure a uniform layer. Subsequently, four additional layers of liquid release agent were applied to reinforce the barrier between the mold and the polymer matrix, thereby facilitating the demolding process after curing. Figure 6 shows the drying stage of the plaster mold. This drying step is critical to reduce residual moisture and surface porosity of the plaster mold, which can otherwise compromise resin flow and promote void formation during vacuum infusion.

To improve the surface quality of the mold, automotive body filler (plastic putty) was applied in order to reduce the intrinsic porosity of the plaster, since the infusion technique employed requires complete sealing of the vacuum chamber.

The procedure for defining the geometry of the motorcycle helmet mold followed the dimensional requirements established in the Brazilian Standard ABNT NBR 7471 [19] for motorcycle safety helmets. The molds were produced using commercial construction plaster, in accordance with the specifications of ABNT NBR 13207 [22], using a mixing ratio of 14 L of water to 20 L of plaster. The mixture was manually applied in successive layers, respecting a maximum curing thickness of 1.5 to 2.5 cm to ensure complete coverage and proper conformity to the model’s geometry.

The coating thickness during mold fabrication was controlled through a combination of volumetric control of the plaster mixture and sequential application of discrete layers. Each plaster batch was prepared with a predefined mass-to-water ratio and applied manually in thin layers, allowing partial setting before the application of the subsequent layer. The thickness of each layer was monitored using a calibrated steel ruler and visual reference marks on the mold box walls, ensuring that the applied thickness remained within the 1.5 to 2.5 cm range. This incremental application approach prevented excessive heat generation during curing, minimized shrinkage and cracking, and ensured proper conformity of the plaster mold to the complex geometry of the helmet model.

Since plaster exhibits high surface porosity after curing, making it unsuitable for use under negative pressure conditions, an initial approach considered shaping the internal profile of the helmet. However, due to the limitations of the available tools and the dimensional variations in the plaster during curing, this alternative was deemed unfeasible.

For the fabrication of the mold intended for resin infusion, parameters such as infusion surface area and vacuum pressure were taken into account. Therefore, wooden boxes were constructed to contain the plaster, with dimensions of 46 × 39 × 15.6 cm^3^ and 45 × 35 × 13.5 cm^3^, corresponding to volumes of 24.3 dm^3^ and 21.3 dm^3^, respectively. The boxes were assembled using 10 mm thick plywood sheets, reinforced externally with metal brackets fixed by screws. The inner edges were sealed with wall putty to minimize potential air leakage during the vacuum process. The box dimensions were defined based on a commercial “street” motorcycle helmet model, designed for urban use.

The external surface of the helmet model received four coats of release wax, followed by mechanical polishing using a wool fiber polishing pad attached to a hand drill, resulting in a smooth and uniform surface finish.

**Figure 7:**
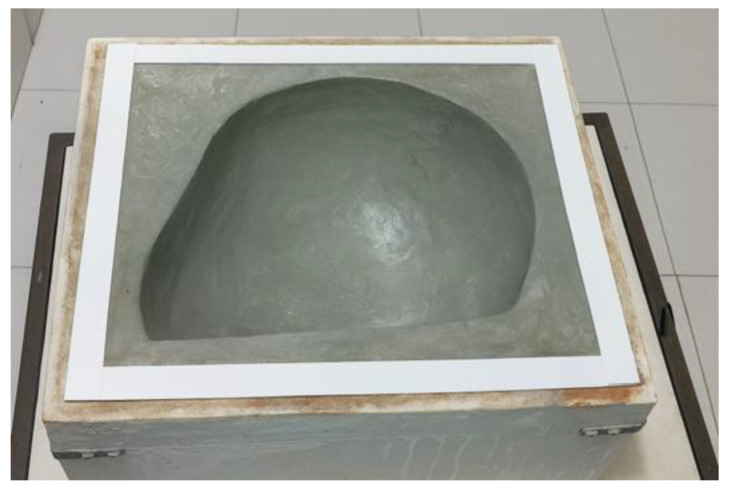
Wooden box to hold the mold.

During the demolding process, small metal spatulas were employed to assist in separating the model from the mold. This operation caused localized damage to the mold surface, requiring repairs after each demolding cycle. The repairs were performed using epoxy putty, selected for its resistance to negative pressure within the infusion chamber. However, such repeated repairs may affect the dimensional accuracy of the final prototype, necessitating careful monitoring and adjustment in subsequent manufacturing steps. Figure 7 shows the wooden box to hold the mold.

#### 2.2.2. Prototype Manufacturing Process

**Figure 8:**
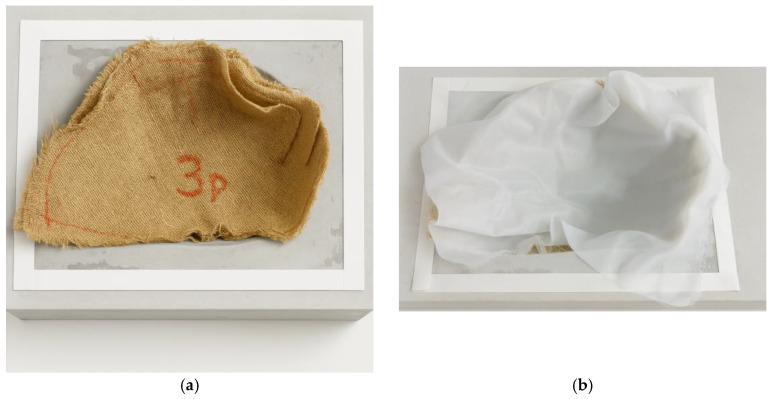

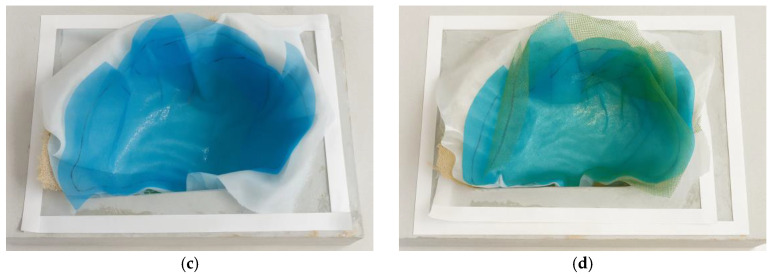
Fabrics used for infusion inside the vacuum chamber: (**a**) Reinforcing fiber, (**b**) Peel ply release fabric, (**c**) Perforated blanket and (**d**) Distributor screen.

The fabrics used in the infusion process must be arranged over the entire surface of the mold to form the composite part to be produced, as shown in Figure 8.

**Figure 9:**
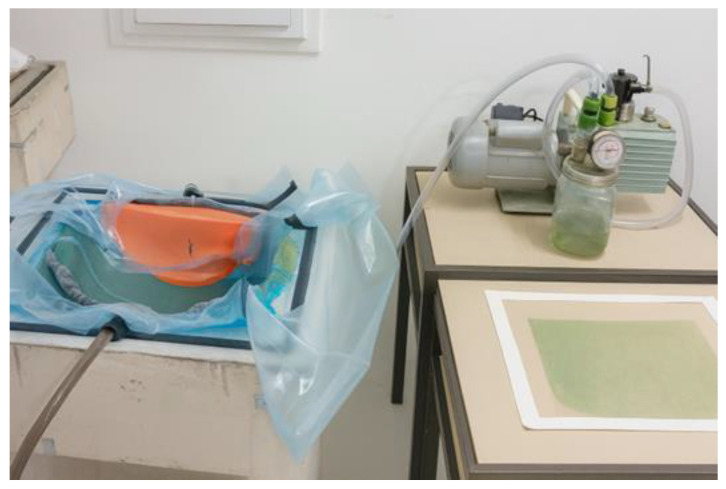
Schematic representation of the vacuum infusion architecture and fiber layup sequence adopted for the helmet shell. The longitudinal axis of the helmet is defined as the front-to-back direction of the shell, extending from the frontal region to the neck opening. The first reinforcement layer was aligned along this axis (0°), while the subsequent layers were arranged at ±45° relative to it to promote balanced load distribution.

The architecture defined to achieve better resin distribution within the vacuum chamber was established through several experimental tests using the resin infusion technique with jute fiber reinforcement. The resin inlet was positioned at the top of the helmet, while the outlet was located at the neck opening. In this configuration, the vacuum brake was positioned vertically, which favored the complete impregnation of the reinforcing fibers and promoted more efficient resin percolation throughout the laminate. The fiber fabrics were arranged in alignment with the longitudinal axis of the helmet, with the first layer oriented at 0°, and each successive layer placed at ±45° relative to the previous one. The longitudinal axis of the helmet corresponds to the front-to-back direction of the shell, extending from the frontal region to the neck opening, as indicated schematically in Figure 9. Specimens were fabricated with two and four reinforcement layers, allowing the evaluation of different stacking configurations on the composite’s structural performance.

**Figure 10:**
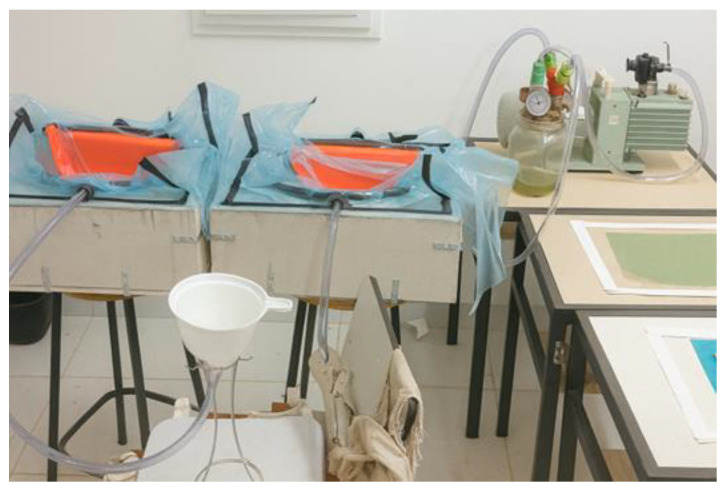
Infusion system.

The materials used in the infusion setup—including the resin distribution and bleed fabrics, the peel ply, and the sealing adhesive tape—were supplied by a local commercial vendor. In addition, polyester resin distribution and absorption meshes were employed to ensure proper resin flow and removal of excess material during the infusion process. The technique used to join the two parts of the prototype produced by the resin infusion process consisted of bonding with the same polyester resin used in the composite matrix, reinforced with jute fiber, effectively performing a resin-based fusion weld between the sections, as shown in Figure 10.

**Figure 11:**
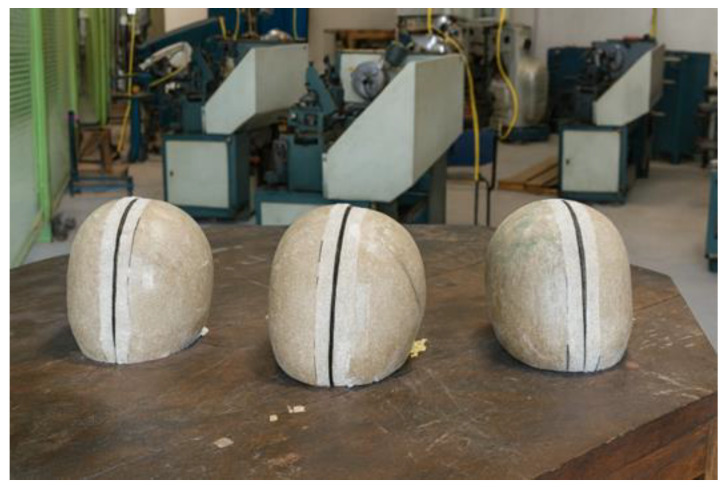
Prototype composite helmet parts made of natural fiber to be glued together.

The infusion system architecture was designed to enable the production of one molded part per stage, allowing the process to yield valuable information regarding composite formation dynamics. This approach made it possible to observe the behavior of the polyester resin as it impregnated and surrounded the natural fiber reinforcement during the infusion process, thereby facilitating the identification and correction of potential defects through the use of an open-mold configuration. Control of fiber impregnation and technological defects during the infusion process was performed through real-time visual monitoring of the resin flow front in an open-mold configuration. Resin filling was carried out gradually under constant vacuum pressure, allowing direct observation of the progression of the resin front and its interaction with the jute fiber reinforcement. This visual control enabled the identification of common infusion-related defects, such as dry spots, air entrapment, race-tracking, and non-uniform wetting. When necessary, corrective actions were applied during the process, including temporary restriction of resin flow, local repositioning or compaction of the reinforcement layers, and adjustment of the resin inlet, in order to promote uniform impregnation and minimize void formation. Figure 11 shows the prototype composite helmet parts made from natural fiber, prior to being bonded together.

**Figure 12:**
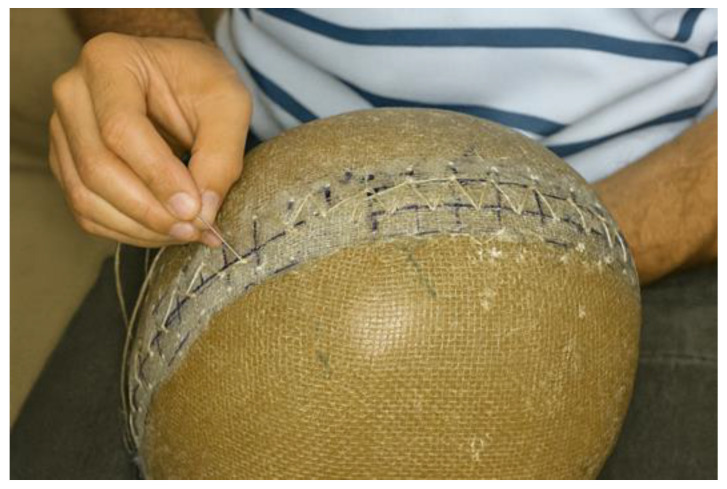
Hand-stitched using the reinforcement fiber itself.

The joining process of the helmet components began with surface preparation, involving sanding and removal of residues from the lateral edges of the composite. This procedure improves interfacial adhesion by eliminating any remaining release agent and impurities that could hinder bonding between the new resin layers. Sanding was performed using a bench drill equipped with a 120-grit sandpaper disc at the Machining Laboratory of the Federal Institute of Pará (IFPA). A recess measuring 20 mm in width and 0.5 mm in depth was machined along the side edges to accommodate the adhesive resin applied between the joined parts. Following surface preparation, holes were drilled along the edges with 10 mm longitudinal spacing and 14 mm diagonal spacing. These geometrical parameters were defined based on preliminary feasibility tests conducted in this study to ensure adequate stitching and joint alignment. These preliminary feasibility tests consisted of exploratory trial-and-error procedures performed on representative composite specimens prior to the fabrication of the helmet prototypes. Different hole spacings and drilling patterns were evaluated to verify stitch ability, edge alignment, ease of manual sewing, and preservation of laminate integrity during drilling and stitching. The selected spacing of 10 mm in the longitudinal direction and 14 mm in the diagonal direction provided an adequate balance between mechanical stability of the joint, alignment accuracy, and minimization of fiber damage and local delamination, and was therefore adopted in the final prototype manufacturing process. A manual stitching procedure was then performed using a hand needle and threads extracted from the same jute fiber used as composite reinforcement, preserving material homogeneity and avoiding the introduction of foreign materials. Drilling was carried out with a 2 mm diameter high-speed steel drill bit, as illustrated in Figure 12.

**Figure 13:**
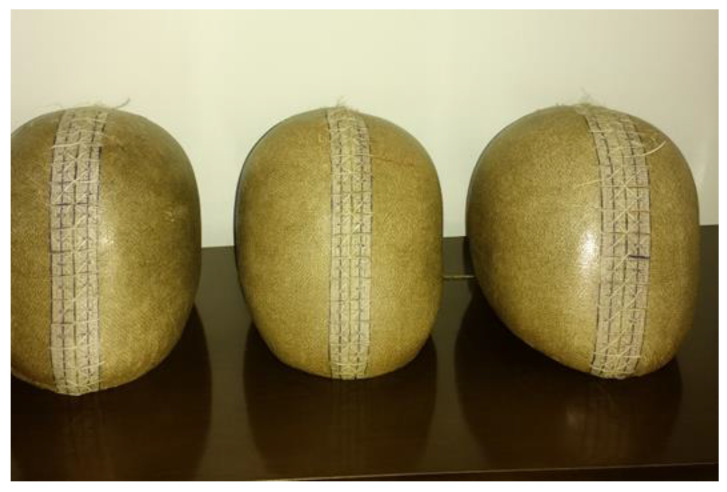
Helmet ready for gasket insertion.

The cross-stitch technique was employed for joining the lateral sections, as it provides greater mechanical stability and uniformity of fixation. The procedure was carried out manually using a hand needle measuring 4.0 cm in length and 1.5 mm in diameter, along with thread obtained from the same jute fiber used as the composite reinforcement. The stitching began by inserting the needle from the outer hole toward the inner side, and then returning in the opposite direction, thereby crossing the threads both internally and externally. This configuration ensured internal locking of the stitch, resulting in a firm and continuous bonding between the edges, as illustrated in Figure 13.

**Figure 14:**
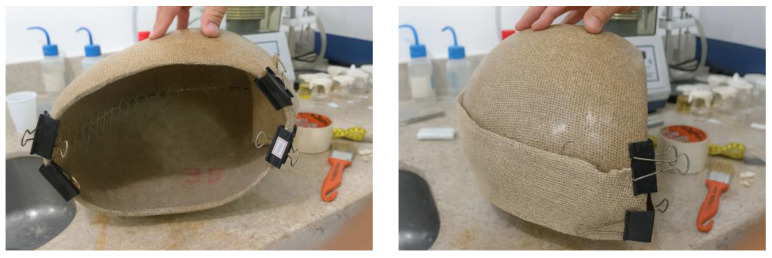
Method of securing the gasket cover using paper clips.

To complete the joining of the helmet’s lateral sections, a reinforcement strip (“gasket cover”) made from the same jute fabric used in the composite laminate was applied to enhance the mechanical strength of the joint region. The width of the gasket cover was defined based on preliminary evaluations performed in this study, aiming to ensure adequate load transfer and to promote a monolithic structural behavior along the bonded interface. Temporary fixation of the gasket cover onto the prototype shell was achieved using metal paper clips, which facilitated correct positioning and conformity of the fabric over the curved surface. This procedure minimized gaps between the shell and the reinforcement strip, improving compaction and resin impregnation during the lamination process with the jute-fiber fabric, as shown in Figure 14.

**Figure 15:**
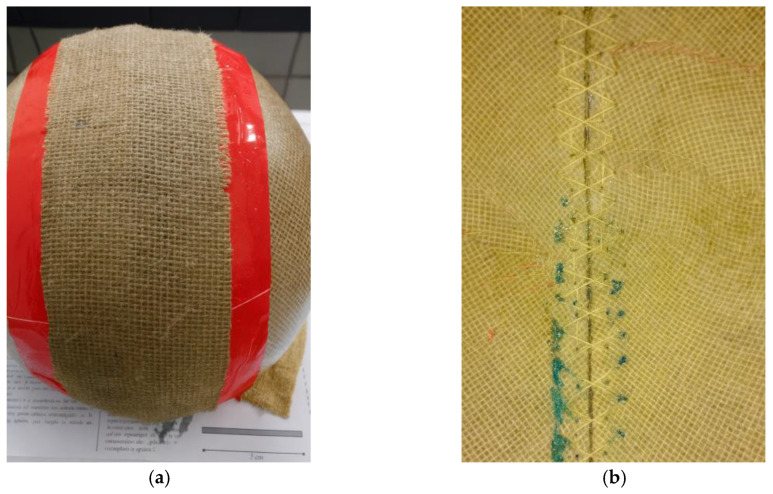
Inner surface of the prototype helmet with joint cover application: (**a**) Resin contained in the double-sided tape, (**b**) Resin impregnated in the stitching holes.

The resin used to bond the joint-cover strip (“gasket cover”) to the helmet shell was an orthophthalic polyester resin, the same material employed in the fabrication of the shell through the infusion process. For the lamination procedure, 150 mL of resin containing 1% methyl ethyl ketone peroxide (MEK-P) catalyst was used, applying the hand lay-up technique to incorporate and secure the joint-cover strip to the main structure. After complete resin curing and removal of the containment elements, it was observed that during lamination, part of the resin penetrated through the thickness of the prototype shell, filling the pre-existing stitching holes. This phenomenon provided an additional reinforcement effect in the joint region, promoting enhanced structural integration between the layers, as illustrated in Figure 15.

**Figure 16:**
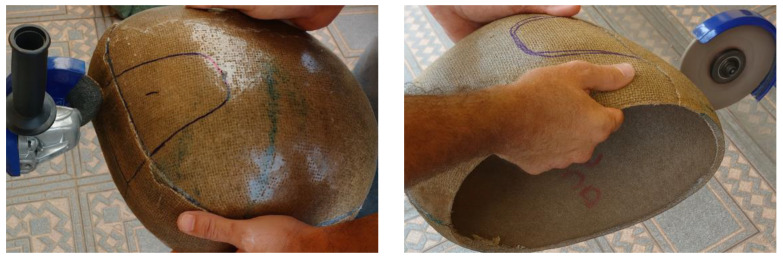
Cutaway view of the visor opening.

After the bonding of the lateral sections of the helmet prototype, it was observed that the complete filling of the stitching holes by the polyester resin, originally made for the passage of the jute thread, resulted in a potential reduction in stress concentration points, thereby decreasing the likelihood of localized failure in this region. The use of the resin infusion technique was deemed unfeasible for this stage, since it involved only a localized reinforcement, which would require the fabrication of a new mold, making the process technically and economically impractical. After a 24-h curing period, the resin containment elements were removed, and the finishing process of the shell was initiated. The opening of the visor area was performed using a hand-held grinder equipped with a cutting disc suitable for resin-based materials. During this operation, appropriate personal protective equipment (PPE) was employed, including safety goggles and a dust mask designed for polymeric materials. The use of protective gloves was avoided due to the smooth surface of the helmet, which could compromise the precision of the cutting line. The cutting path was guided by a reference marking obtained from a commercial helmet, which provided all necessary dimensional parameters for the prototype fabrication, as illustrated in Figure 16.

The cutting of the visor opening was an essential step to ensure that the dynamic measurements obtained from the prototype would yield results comparable to those of commercial helmets currently available on the market. After completing the cut, the surface finishing was performed using a bench drill located at the Mechanical Laboratory of the Federal Institute of Pará (IFPA), operating at 2300 rpm and fitted with a rotary sanding adapter with 120-grit sandpaper. During this stage, all burrs and irregularities resulting from the manufacturing process were removed, producing a surface with the required finish for conducting impact absorption and lateral compression tests. The geometry adopted for the prototype demonstrated that the polyester resin infusion process reinforced with natural fibers is a viable manufacturing technique for producing elongated and geometrically complex structures, meeting the quality standards required by current industry applications. The development of the helmet prototype was based on the infusion of orthophthalic polyester resin, using a dual-stage vacuum pump (530 W, 220 V) available at the Resin Infusion Laboratory of the Federal Institute of Education, Science and Technology of Pará (IFPA), with a maximum vacuum capacity of 1 atm (760 mmHg). During the plate infusion process, it was observed that resin loss could be reduced through optimization of the initial infusion system architecture. Consequently, an improved setup was adopted, allowing for better control of the resin flow and the gelation transition. The moment of catalyst addition (1% MEK-P) to the polyester resin was considered as time zero (t_0_), providing an effective working time of approximately 10 min before the onset of gelation—a critical factor for the successful completion of the process.

### 2.3. Characterization of the Composite Bond

To characterize the bonding between the helmet components, polyester resin composite plates were produced by resin infusion, maintaining the same fiber arrangement and orientation used in the prototype. From these plates, containing two and four layers of jute fabric, tensile test specimens were extracted in accordance with the ASTM D3039 [18] standard. Four different specimen configurations were prepared: specimens without joints, representing the intact material; specimens stitched with jute thread, simulating manual suturing; specimens bonded using a jute fabric joint-cover strip; and specimens stitched with jute thread and reinforced with a bonded jute fabric joint-cover strip. This experimental approach enabled a comparative evaluation of the mechanical performance of each joining configuration, providing insights into the influence of the bonding technique on the tensile strength of the composite material.

#### 2.3.1. Tensile Tests of the Connection

**Figure 17:**
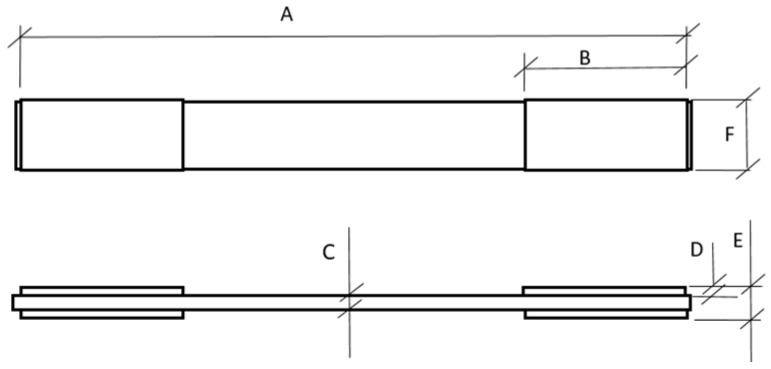
Dimensions of test specimens for tensile testing.

The tensile test specimens were fabricated from composite plates produced by the polyester resin infusion method, reinforced with woven jute fiber fabric. For each configuration, a minimum of five specimens was prepared to ensure statistical reliability of the results. The tests were conducted using an AROTEC WDW 100E universal testing machine, equipped with a 10 kN load cell and operating at a crosshead speed of 5 mm/min, at the Federal Institute of Pará (IFPA). For all tensile specimens, the 0° direction was defined along the same longitudinal reference used for the helmet shell, corresponding to the front-to-back axis of the structure. All tensile tests were performed in accordance with the ASTM D3039 [18] standard, following the standardized specimen dimensions schematically illustrated in Figure 17 and listed in Table 1.

In this study, the term elongation at rupture (ΔLrup) refers to the absolute elongation measured within the gauge length of the specimen at the moment of failure, expressed in millimeters. This parameter represents the total axial deformation accumulated during tensile loading and should not be confused with crosshead displacement. The corresponding tensile strain (εrup) can be directly obtained by normalizing ΔLrup by the initial gauge length, in accordance with ASTM D3039 [18].

**Figure 18:**
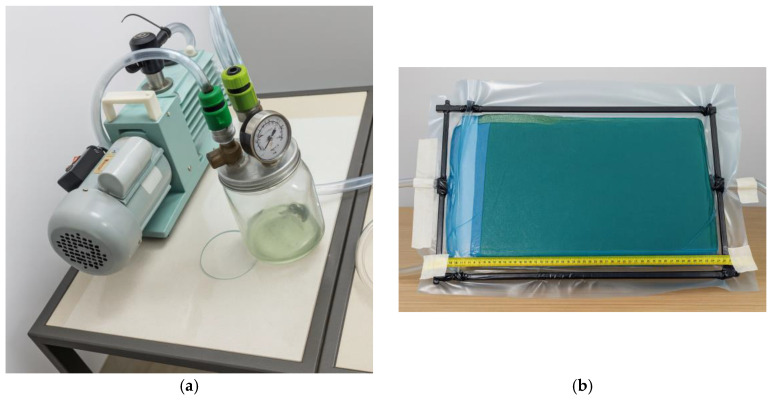
(**a**) Vacuum pump, (**b**) Composite plate produced by resin infusion.

The composite plates reinforced with woven jute fabric, containing two (2) and four (4) reinforcement layers, were manufactured using the polyester resin infusion process under a vacuum pressure of 760 mmHg. The fabrication was carried out on a flat glass surface measuring 330 mm × 550 mm, ensuring uniform thickness and surface quality. From these plates, a total of 20 tensile specimens were obtained, with average thicknesses of 2.0 mm for the two-layer plates and 3.6 mm for the four-layer plates, as illustrated in Figure 18. The laminate thickness was not controlled by tooling but resulted from the vacuum infusion process, leading to small variations typical of this manufacturing route, which were quantified and reported as average values with tolerances.

**Figure 19:**
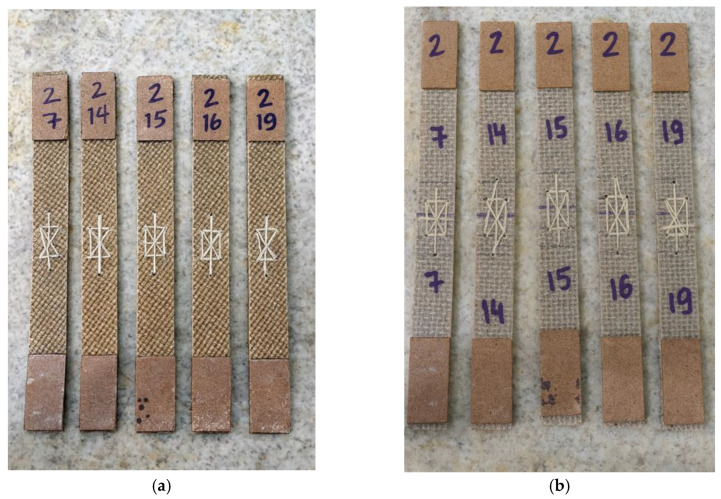
Test specimen with 2 layers of reinforcement and stitched with jute thread. (**a**) Front, (**b**) Back.

**Figure 20:**
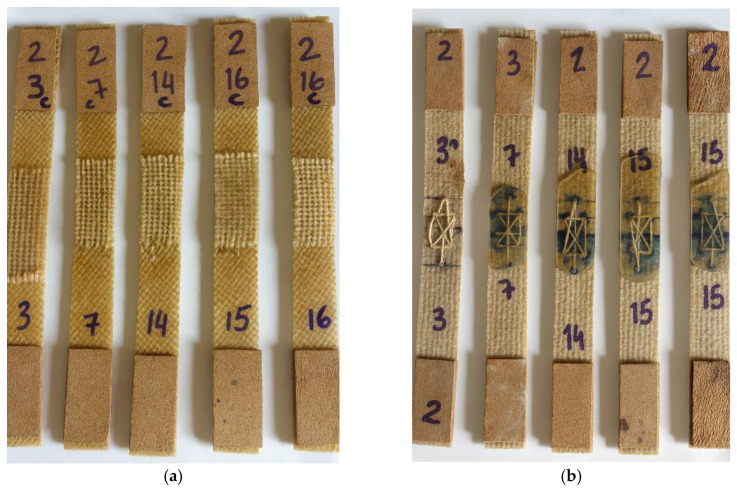
Test specimen with 2 layers of jute fiber reinforcement, stitched with jute thread and glued with backing tape. (**a**) Front, (**b**) Back.

**Figure 21:**
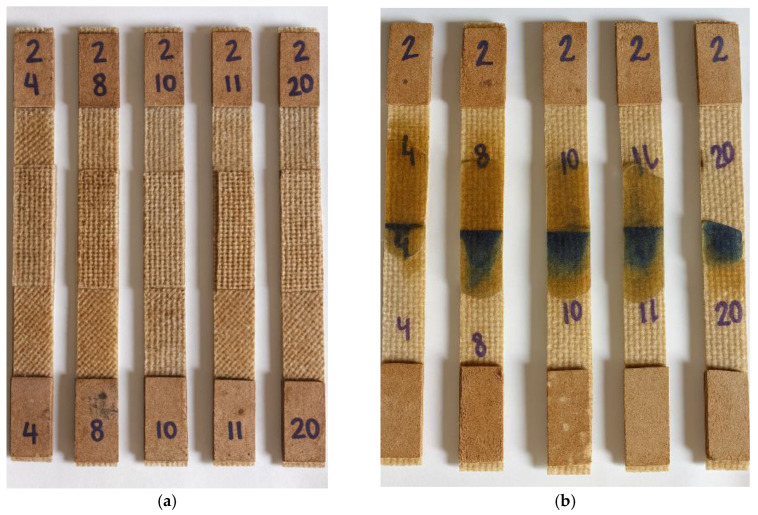
Test specimen with 2 layers of jute fiber reinforcement, bonded with backing tape. (**a**) Front, (**b**) Back.

**Figure 22:**
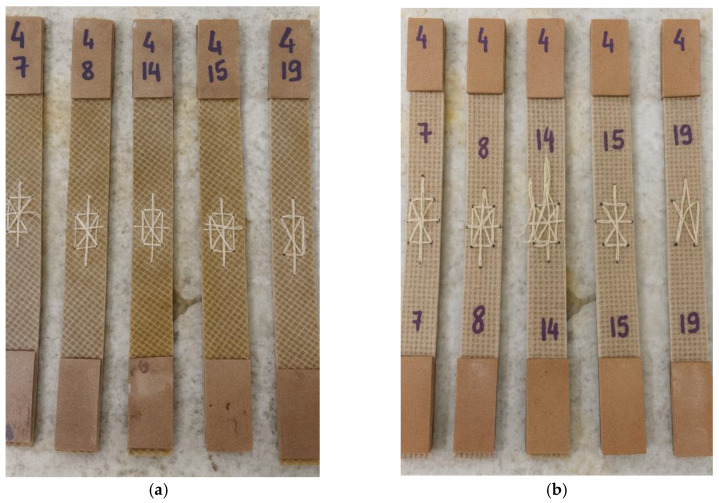
Test specimen with 4 layers of jute fiber reinforcement, stitched with jute thread. (**a**) Front, (**b**) Back.

**Figure 23:**
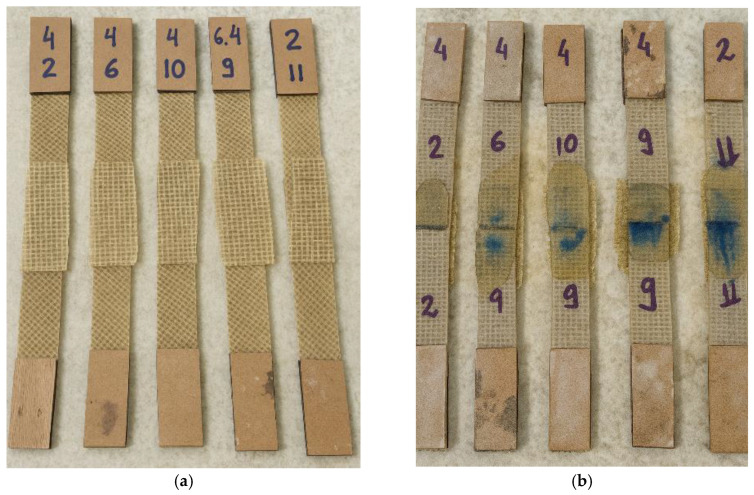
Test specimen with 4 layers of jute fiber reinforcement, bonded with backing tape. (**a**) Front, (**b**) Back.

**Figure 24:**
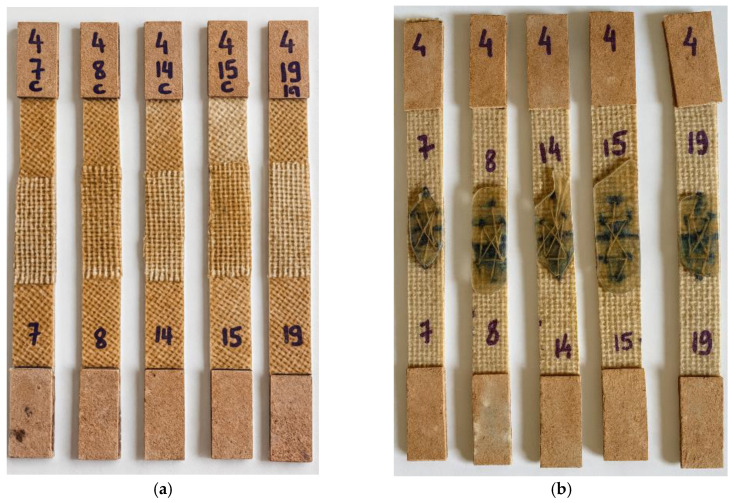
Test specimen with 4 layers of jute fiber reinforcement, stitched with jute thread and glued with backing tape. (**a**) Front, (**b**) Back.

The tensile test specimens were extracted from the composite plates produced by polyester resin infusion using a cutting disc mounted on an angle grinder, ensuring that the material integrity was preserved and that the specimens remained suitable for tensile testing. Four different types of specimens were prepared, each representing a distinct joining configuration: unjoined specimens, representing the intact material; stitched specimens, joined using jute thread; joint-cover specimens, bonded with a jute fabric strip; and stitched joint-cover specimens, combining both stitching and bonding techniques. These specimen configurations are illustrated in Figure 19, Figure 20, Figure 21, Figure 22, Figure 23 and Figure 24.

Before testing, all specimens were visually and dimensionally inspected to ensure uniformity and structural integrity. Specimens exhibiting dry spots, delamination, edge defects or failure initiation outside the gauge section were excluded from the analysis, representing less than 10% of the total. The observed variability in the mechanical results is primarily attributed to subtle differences inherent to the vacuum infusion process, including local variations in resin flow, void content, and effective laminate thickness. Such heterogeneities are typical of natural-fiber composites, where the architecture and surface morphology of jute fibers can promote localized resin-rich or resin-deficient regions. The remaining specimens showed consistent load–displacement behavior and similar fracture patterns, confirming the reliability of the final dataset used for mechanical characterization.

Therefore, the overall dispersion of the results can be considered acceptable and consistent with the manufacturing process employed. The findings indicate that the vacuum infusion technique ensured adequate laminate consolidation and reproducibility, with only minor variations attributable to intrinsic characteristics of natural-fiber reinforcement and process-induced microstructural effects.

**Figure 25:**
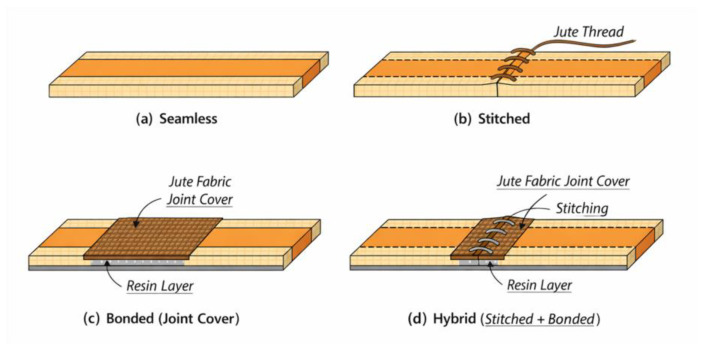
Schematic representation of the tensile specimen joining configurations: (**a**) Seamless specimen without joint; (**b**) Stitched joint using jute fiber thread; (**c**) Bonded joint with jute fabric joint-cover strip; (**d**) Hybrid joint combining stitching and bonded joint-cover. Schematics are not to scale and are intended to illustrate the joining concepts adopted in this study.

To improve clarity and facilitate the understanding of the different joining strategies adopted in this study, a schematic representation of each tensile specimen configuration is presented in Figure 25. The schematics illustrate the main geometric features and joining mechanisms associated with the seamless, stitched, bonded (joint cover), and hybrid (stitched + bonded) configurations.

#### 2.3.2. Prototype Compression Tests

**Figure 26:**
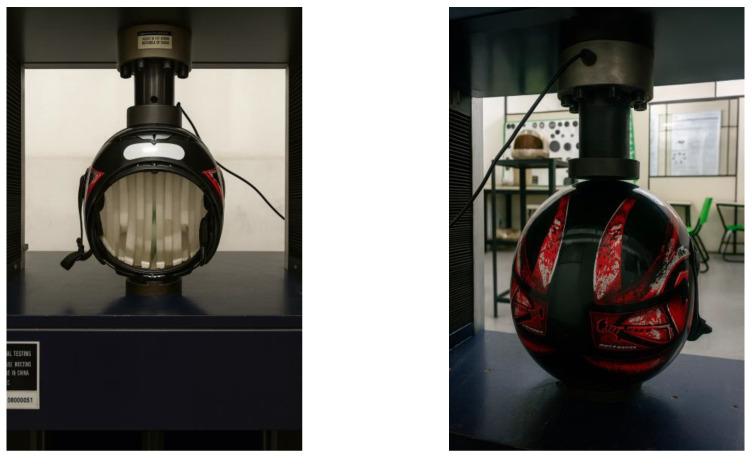
Frontal compression test on a commercial helmet.

**Figure 27:**
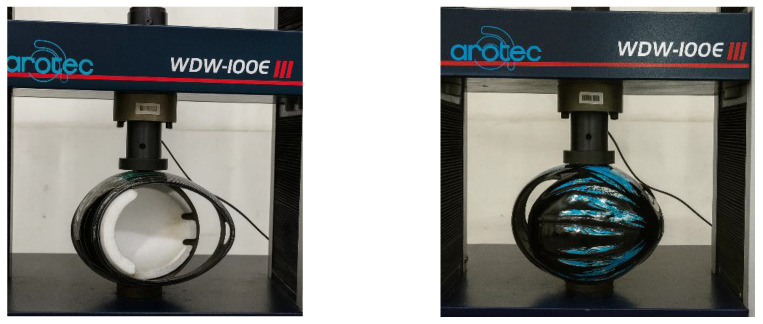
Lateral compression test on a commercial helmet.

According to the standard requirements, the helmet under testing must not exhibit a deformation greater than 40 mm in either the longitudinal or transverse directions, nor a permanent deformation exceeding 15 mm after returning to its initial condition. In addition, no cracks or fissures should be observed in the helmet shell. The compression tests were carried out at the Mechanical Testing Laboratory of the Federal Institute of Pará (IFPA). Initially, reference tests were performed using a commercial helmet without reinforcement, in accordance with the ABNT NBR 7471 [19] standard, as shown in Figure 26 and Figure 27.

**Figure 28:**
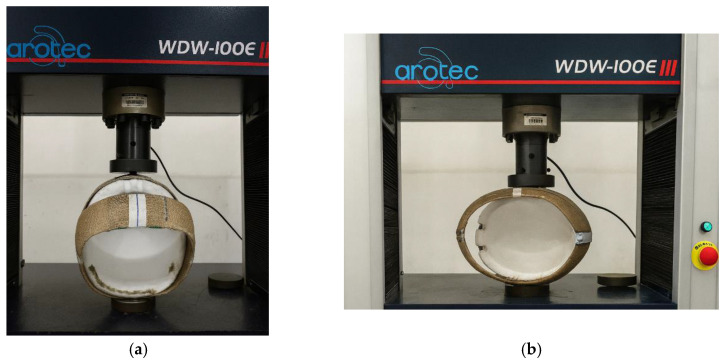
Compression test on a helmet with 2 layers of natural jute reinforcement. (**a**) Front, (**b**) Side.

**Figure 29:**
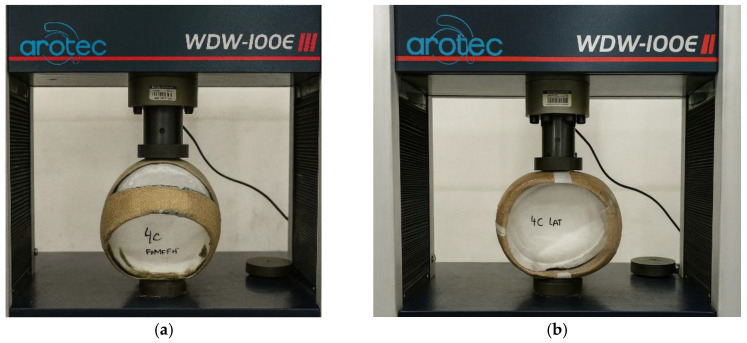
Compression test on a helmet with 4 layers of natural jute reinforcement. (**a**) Front, (**b**) Side.

After performing the frontal and lateral compression tests on different commercial helmets in accordance with ABNT NBR 7471 [19], the same procedure was applied to the composite helmet prototypes made of polyester resin reinforced with natural jute fabric, as illustrated in Figure 28 and Figure 29. The prototypes used in the compression tests were manufactured with two (2) and four (4) layers of jute fabric arranged at ±45° to form a balanced laminate. The ±45° fiber orientation adopted for the helmet prototypes was defined with respect to the longitudinal axis of the helmet, which corresponds to the front-to-back direction of the shell. This same longitudinal axis was used as the 0° reference direction for the fabrication and testing of the tensile specimens according to ASTM D3039 [18]. As a result, both the flat test coupons and the helmet shells share a consistent fiber orientation reference system, ensuring that the mechanical response observed at the material level is directly comparable to the structural behavior of the full-scale prototypes. The use of a balanced ±45° layup further ensured quasi-isotropic in-plane behavior and minimized directional bias in the compression response of the helmet shells.

### 2.4. Statistical Analysis

The statistical evaluation of the experimental data was conducted to determine the significance of differences among the tested configurations and laminate thicknesses. All numerical results obtained from the tensile and compression tests were subjected to inferential analysis using parametric and post-hoc procedures consistent with the experimental design. A two-way analysis of variance (ANOVA) was applied to the tensile data to evaluate the main effects of Layer (2 or 4) and Configuration (Seamless, Stitched, Joint Cover, and Joint + Stitched), as well as the interaction between these factors. For the compression tests, both frontal and lateral, the ANOVA was performed considering Layer (2 or 4) and Material type (composite or commercial helmet) as independent factors. In all cases, statistical significance was established at a confidence level of α = 0.05. Following the ANOVA, Tukey’s Honest Significant Difference (HSD) test was conducted to identify which pairs of groups differed significantly. The HSD values were used as a reference threshold for determining the minimum significant difference between mean values. These procedures allowed a detailed comparison of the influence of laminate thickness and joint configuration on the mechanical performance of the composites, ensuring that the observed trends were supported by statistically significant evidence. Prior to conducting the ANOVA, the data were examined for compliance with assumptions of normality and homogeneity of variances using the Shapiro–Wilk and Levene’s tests, respectively.

## 3. Results and Discussion

### 3.1. Tensile Test Analysis

Table 2 presents the mean values, standard deviations, and coefficients of variation obtained in the tensile tests of polyester/jute composites, considering different construction configurations and number of layers.

In general, it is observed that the mechanical performance of laminates varies significantly depending on the presence of seams, joints, and the thickness of the laminate, reflected in the number of layers used.

Seamless composites showed the best overall performance among all configurations evaluated. For two layers, the maximum force (Qrup) reached 0.80 kN, with a displacement at rupture (Δlrup) of 2.51 mm. When the number of layers increased to four, the maximum force doubled to 1.60 kN, while the displacement decreased slightly to 2.21 mm, demonstrating an increase in structural stiffness. This behavior is typical of thicker laminates, in which the continuity of the fibers in the tensile direction allows for more efficient load transfer, resulting in greater strength and lower overall deformability. Furthermore, the coefficients of variation obtained for the seamless laminates indicate good manufacturing consistency of the lamination process. For the maximum load (Qrup), the coefficient of variation was as low as 4.4%, while for the elongation at rupture (ΔLrup) it reached 9.2%. Although the latter is not the lowest value measured among all configurations, coefficients of variation below approximately 10–12% are widely considered acceptable for natural-fiber-reinforced polymer composites, given the intrinsic variability associated with fiber morphology, moisture content, and resin impregnation. Therefore, the observed dispersion levels indicate a satisfactory degree of reproducibility for vacuum-infused jute fiber laminates.

On the other hand, stitched composites showed the lowest strength values, with Qrup of 0.12 kN for two layers and 0.13 kN for four layers. Although the elongation at rupture (ΔLrup) increased from 1.19 mm to 1.55 mm, this behavior should not be interpreted as an improvement in damage tolerance. Instead, the higher elongation is associated with reduced load-bearing capacity and a more compliant response, reflecting lower structural strength and an apparent increase in deformability resulting from fiber discontinuity and localized damage introduced by the stitching process. This is due to the fact that the stitching process breaks fibers and creates resin-rich micro zones, which act as stress concentrators and reduce the load-bearing capacity of the laminate. Still, the slight increase in displacement suggests that stitching provides some delamination containment, promoting more progressive failures.

Although no microscopic fractographic analysis was conducted in the present study, the proposed failure mechanisms are supported by consistent macroscopic fracture patterns observed after testing and by well-established micromechanical behavior reported for natural-fiber-reinforced polyester composites. In stitched specimens, the introduction of stitching holes inherently disrupts fiber continuity, promoting localized stress concentration and premature fiber breakage, as widely reported in the literature. Additionally, the local accumulation of resin around the stitching region during vacuum infusion is expected to generate resin-rich zones, which act as preferential sites for crack initiation. Similar mechanisms have been reported for bonded overlap regions, where thickness mismatch and interfacial resin layers contribute to non-uniform stress distribution and early failure. These interpretations are consistent with the observed mechanical response and with previous studies on natural and synthetic fiber composite joints.

Samples with overlapping joints showed anomalous and concerning behavior. Contrary to expectations, increasing the number of layers resulted in reduced strength and deformation. For two layers, Qrup was 0.74 kN, with Δlrup of 1.56 mm; however, with the addition of two more layers, these values dropped to 0.69 kN and 0.86 mm, respectively. This performance loss suggests that the presence of thick joints and overlapping regions created stress peaks and discontinuities in the structure, compromising load transfer between layers. Possibly, the increased thickness intensified the occurrence of voids and resin-rich regions, which act as weak points and promote premature laminate failure. Furthermore, the relatively high coefficient of variation (11.6%) for displacement at rupture indicates low process stability, reinforcing the hypothesis of inconsistencies in joint formation.

In contrast, the behavior of the samples combining joint and stitching was notably superior, especially in the four-layer laminate. In this configuration, the maximum force reached 1.43 kN and the displacement 1.80 mm, with low coefficients of variation (5.6% and 6.1%). This result demonstrates that the combination of stitching and jointing promotes structural synergy, simultaneously increasing strength and containing delamination. The stitching, in this case, contributes to restricting delamination between the overlapping layers, while the greater number of layers ensures a more efficient fiber network for load transfer. The observed behavior reflects a more progressive and less catastrophic failure mode, which is desirable in components subjected to complex stresses and impacts, such as helmets and protective structures.

This marked increase in Qrup for the hybrid configuration is not the result of a simple additive effect of stitching and joint cover, but rather of a synergistic interaction between both joining mechanisms. In stitched-only specimens, the sewing holes locally disrupt fiber continuity and introduce stress concentrations and resin-rich regions, leading to premature failure and low Qrup values (0.12–0.13 kN). In joint-cover specimens, load transfer occurs mainly through the bonded overlap; however, failure is typically governed by interfacial debonding and delamination driven by peel stresses at the overlap edges, resulting in intermediate Qrup values (0.69–0.74 kN). When both techniques are combined, the joint cover provides the primary load-transfer path (bridging effect), while stitching acts as a through-thickness mechanical constraint that limits interfacial crack propagation and delays delamination growth. This interaction promotes a more progressive failure mode and explains the significantly higher Qrup values observed for the joint + stitched specimens. The effect is particularly pronounced in the four-layer laminate, where the increased thickness and load-bearing cross-section reduce the relative severity of stitching-induced damage and enhance the effectiveness of through-thickness reinforcement.

Further qualitative analysis can be performed considering the product Qrup × Δlrup, used as a simplified estimate of the energy absorbed until rupture. In this context, the Seamless 4-layer structure showed the highest value (approximately 3.54 kN·mm), followed by the Joint + stitched 4-layer structure (2.57 kN·mm), reinforcing the superiority of these two configurations. The others showed progressively lower performance, with the Stitched 2-layer structure being the least efficient (0.14 kN·mm). Thus, it is verified that structures without discontinuities (Seamless) or with optimized seams (Joint + stitched) are the most suitable for applications requiring high load capacity and good energy dissipation. Although the synergistic effect of the hybrid joint is consistent with established joint mechanics, a detailed fracture-mode mapping and microstructural analysis would further quantify the relative contributions of debonding, delamination, and through-thickness reinforcement.

**Figure 30:**
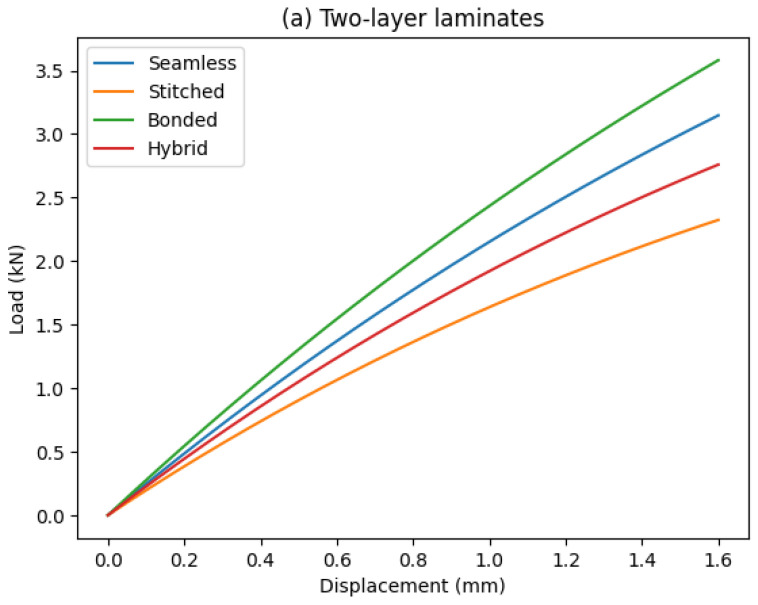

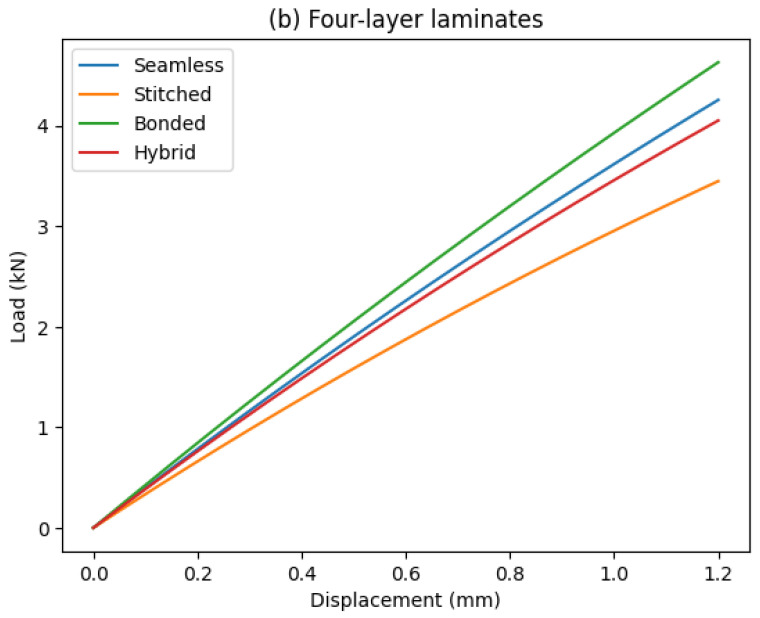
Load–displacement curves obtained from tensile tests ASTM D3039 [18] for polyester/jute composites with different joining configurations: seamless, stitched, bonded (joint cover), and hybrid (joint cover + stitching). (**a**) Two-layer laminates; (**b**) Four-layer laminates.

In order to provide a more comprehensive assessment of the mechanical response of the polyester/jute composites, the load–displacement curves obtained from the tensile tests are presented in Figure 30. These curves allow a direct comparison of stiffness, load-bearing capacity, and deformation behavior among the different joining configurations and laminate thicknesses, complementing the quantitative results summarized in Table 2.

Figure 30 shows the representative load–displacement curves obtained from the tensile tests for two- and four-layer laminates with different joining configurations. For both laminate thicknesses, seamless specimens exhibited the highest initial stiffness and a nearly linear response up to failure, reflecting efficient load transfer due to uninterrupted fiber continuity. Stitched specimens presented the lowest stiffness and load-bearing capacity, associated with fiber disruption and stress concentration around the stitching holes.

Bonded specimens showed intermediate behavior, with higher stiffness than stitched joints but lower than seamless laminates, indicating that load transfer was governed by the bonded overlap region and affected by interfacial stress concentrations. The hybrid configuration (joint cover + stitching) exhibited a more progressive mechanical response, combining relatively high stiffness with increased displacement prior to rupture, particularly in the four-layer laminate. This behavior suggests improved damage tolerance and enhanced interlaminar constraint, as stitching limits delamination propagation while the bonded joint contributes to effective load redistribution.

### 3.2. Compression Test Analysis

The results obtained for the compression test, performed according to ABNT NBR 7471 [19] with load levels ranging from 30 to 630 N (Table 3), allow a detailed analysis of the mechanical behavior of three distinct systems: the two-layer composite, the four-layer composite, and the commercial material used as a reference. It is important to note that, according to ABNT NBR 7471 [19], compression tests were performed under controlled load increments up to a maximum value of 630 N. The objective of the test is to evaluate deformation and structural integrity rather than induce catastrophic failure. Therefore, the maximum compressive load (Pmax) reported in Table 3 and Table 4 corresponds to the maximum applied load prescribed by the standard and not to the ultimate failure load of the helmet structures.

In all cases, the frontal displacement increased progressively with the applied load, which is expected in typical linear-elastic behavior up to a certain limit. However, the rate of increase in the displacement, that is, the apparent stiffness of the system, varied significantly between the samples, revealing important differences in the ability to resist deformation under compression.

The two-layer composite exhibited the most deformable behavior, showing the largest displacements at all load levels. Between 30 and 630 N, the displacement increased from 1.11 mm to 15.09 mm, indicating a total variation of approximately 13.98 mm. This high deformability reflects lower structural stiffness, with an estimated average stiffness of around 42.9 N/mm. This behavior suggests that the two-layer structure has a greater capacity to accommodate deformations, either due to its smaller thickness, lower density of reinforcement interfaces, or the existence of micro voids and irregularities that compact under load. It is possible that the composite exhibits gradual stiffening as the load increases, a common phenomenon in porous or partially compacted structures.

On the other hand, the four-layer composite exhibited the stiffest behavior among the three analyzed. Its displacement ranged from 0.66 mm under 30 N to 9.97 mm under 630 N, corresponding to a total deformation of 9.31 mm and an average stiffness of approximately 64.5 N/mm. The response is practically linear throughout the entire loading range, demonstrating a more stable internal structure and better load transfer between the layers. This behavior is typical of well-consolidated materials, with fewer interlaminar defects and good adhesion between the matrix and the reinforcement. Thus, increasing the number of layers results in a substantial improvement in compressive strength and less susceptibility to permanent deformation.

The higher stiffness observed for the four-layer laminates can be attributed to mechanisms commonly associated with increased laminate thickness, such as improved stress redistribution across the thickness and enhanced interlaminar constraint. Although no microstructural analysis was performed in the present study, previous investigations have shown that thicker laminates tend to exhibit lower susceptibility to interlaminar defects, reduced stress concentration at ply interfaces, and more uniform load transfer between layers. These effects are associated with a higher number of load-bearing interfaces and improved confinement of deformation, which limits local interlaminar sliding and delamination initiation [4,6,20]. Similar trends have also been reported for natural-fiber-reinforced composites manufactured by vacuum-assisted processes, where increased laminate thickness contributes to improved consolidation and reduced sensitivity to local voids and resin-rich regions [7].

The commercial material exhibited intermediate behavior between the two composite configurations. Its displacement increased from 1.00 mm to 10.76 mm between 30 and 630 N, indicating a total deformation of 9.76 mm and an average stiffness of approximately 61.5 N/mm. However, its response curve shows a higher initial stiffness in the early loading stages, followed by a slight reduction as the load increases. This behavior suggests that the commercial material, possibly denser and more homogeneous, is already partially compacted from the beginning of the test, exhibiting little initial deformation. As the load increases, structural rearrangements, microcracks, or internal micro shears occur, slightly reducing its incremental strength and explaining the observed softening tendency.

The commercial helmet exhibited higher initial stiffness at the lower load levels, followed by a slight softening trend as the applied load increased. This behavior may be attributed to a combination of microstructural and manufacturing-related factors. Commercial helmets are typically produced under high-pressure molding or compression processes, which promote a high degree of initial compaction and reduced void content, resulting in a stiffer response at the early stages of loading. As the compressive load increases, however, the onset of microcracking in the polymer matrix, local interfacial debonding between reinforcement and matrix, or the gradual closure and rearrangement of residual internal porosity may occur, leading to a reduction in incremental stiffness.

Additionally, pre-compaction of the commercial material during manufacturing may cause the structure to respond elastically at low loads, followed by progressive damage accumulation or microstructural reorganization at higher load levels. These mechanisms are commonly reported in polymer-based helmet shells and thermoset composite structures subjected to quasi-static compression and are consistent with the observed softening tendency in the present results.

Comparatively, at all load levels, the two-layer composite showed the greatest displacements—approximately 1.4 to 1.5 times greater than those observed in the four-layer composite and the commercial material. At the maximum load of 630 N, the displacement of the two-layer material (15.09 mm) was about 51% greater than that of the four-layer material (9.97 mm) and 40% greater than that of the commercial material (10.76 mm). The difference between the four-layer composite and the commercial material, although small, is consistent: the four-layer composite showed slightly smaller displacements throughout the entire range, demonstrating greater stiffness and dimensional stability.

The results are also consistent with the behavior predicted by the ABNT NBR 7471 [19] standard, which recommends testing at increasing load levels to evaluate deformation under compression and minimize the effects of initial settlement. The first level of 30 N likely corresponds to a settlement preload, and it is natural to observe a greater variation in displacement at this stage. From the second level onwards, the curves tend to stabilize, which reinforces the importance of considering only the linear region of the curve for calculating the effective stiffness of the material.

From a micromechanical point of view, the more deformable behavior of the two-layer samples can be attributed to their smaller thickness and reduced number of reinforcement interfaces, which favors the compression of internal microstructures and the rearrangement of fibers under load. The four-layer samples, on the other hand, exhibit better stress redistribution and less interlaminar flexibility, resulting in greater stiffness and a better ability to withstand compression without excessive deformation. The commercial material, in turn, appears to exhibit a more compact and homogeneous structure, reacting with greater stiffness at the beginning of loading, but showing small incremental strength losses with increasing load, possibly due to internal microdamage.

In practical terms, if the project’s objective is to achieve greater dimensional stability and compressive strength, the four-layer composite is clearly the best choice, surpassing the performance of the commercial material. If the focus is on absorbing deformations or damping stresses, the two-layer composite proves more suitable, as it withstands greater displacements without abrupt failure. The commercial material, in turn, represents an intermediate alternative, balancing stiffness and deformation capacity.

The results obtained for the compression test at increasing load levels, ranging from 30 N to 630 N according to the procedures established by the ABNT NBR 7471 [17] standard (Table 4), allow a detailed analysis of the lateral deformation behavior of the three systems evaluated: the two-layer composite, the four-layer composite, and the commercial material used as a reference.

In all cases, it was observed that the lateral displacement increased progressively with the applied load, which is expected in materials subjected to compression. However, the rate of increase in displacement, which reflects the lateral stiffness of the system, varied significantly between the samples, highlighting structural and stability differences between the materials analyzed.

The two-layer composite exhibited the greatest lateral displacements at all load levels, indicating it to be the most transversely deformable material. The displacement increased from 0.83 mm under 30 N to 13.73 mm under 630 N, resulting in a total variation of 12.90 mm and an average stiffness of approximately 46.5 N/mm. This behavior reflects a more flexible structure, with less interlaminar restraint and greater susceptibility to shear deformation. It is likely that the material possesses micro voids and small irregularities that progressively accommodate themselves as the load increases, which explains the gradual stiffening observed after the first load levels. This trend is typical of composites with smaller thickness and less structural confinement, in which internal compaction and fiber rearrangement contribute to an increase in stiffness as the test progresses.

The four-layer composite, in turn, exhibited the smallest lateral displacement across the entire loading range, demonstrating a much more stable and rigid behavior. The displacement varied from 0.35 mm to 7.24 mm between the first and last level, corresponding to a total variation of 6.89 mm and an estimated average stiffness of 87.1 N/mm, almost double that obtained for the two-layer composite. This result indicates a structure with excellent interlaminar locking, good fiber-matrix adhesion, and the ability to resist transverse deformation. The response was practically linear throughout the load range, suggesting that the four-layer laminate has a more uniform internal stress distribution and that the layers work in an integrated manner, without exhibiting significant slippage or delamination between them. This behavior is characteristic of well-consolidated composites, in which structural integrity is maintained even under high compression.

The commercial material exhibited intermediate behavior between the two composite configurations. Lateral displacement increased from 1.19 mm to 11.47 mm in the range of 30 to 630 N, corresponding to a total variation of 10.28 mm and an average stiffness of approximately 58.4 N/mm. However, it is observed that the initial displacement was high even in the first stage, suggesting that the material underwent a process of geometric or structural accommodation in the initial stages of the test, possibly due to the presence of internal gaps, pre-existing microcracks, or slight eccentricity in the load application. After this initial phase, the material exhibited continuous stiffening behavior, with less displacement increase in subsequent stages, indicating progressive compaction of the microstructure and closure of any internal discontinuities.

A direct comparison between the materials shows that the four-layer composite exhibited the best performance in terms of lateral stability, followed by the commercial material, and finally the two-layer composite, which showed the highest deformability. Considering the maximum displacement under 630 N, the four-layer laminate deformed approximately 47% less than the two-layer laminate and approximately 37% less than the commercial material. This difference is significant and confirms that increasing the number of layers substantially improves the stiffness and the composite’s ability to resist lateral compression.

The comparative analysis of the two- and four-layer laminates revealed substantial relative differences in both stiffness and deformation parameters. In the frontal compression test, the stiffness increased from 42.9 to 64.5 N/mm, corresponding to a 50.3% rise in rigidity when the laminate thickness was doubled. Similarly, in the lateral compression test, the stiffness exhibited an even more pronounced improvement, increasing from 46.5 to 87.1 N/mm—an 87.3% gain in lateral rigidity. These results clearly demonstrate that increasing the number of layers significantly enhances the composite’s ability to resist compressive loads in both directions.

Regarding deformation at the maximum load (630 N), the effect of laminate thickness was inverse: the frontal deformation decreased from 15.09 mm to 9.97 mm, representing a 33.9% reduction, while the lateral deformation decreased from 13.73 mm to 7.24 mm, equivalent to a 47.3% reduction. This reduction in displacement indicates a more stable structural response, reflecting improved interlaminar confinement and reduced compressibility of the thicker laminates.

A similar trend was observed in the tensile tests ASTM D3039 [18]. The increase from two to four layers led to a 100% gain in maximum load (Qrup) for the seamless configuration and an 81% gain for the hybrid joint + stitched configuration. In contrast, the stitched configuration exhibited only a slight improvement (≈8%), whereas the joint cover configuration showed a small reduction (≈7%) in tensile strength. These results highlight that the benefits of increased thickness are strongly dependent on the joint type: the seamless and hybrid configurations take full advantage of additional reinforcement layers, while discontinuous joints do not exhibit the same proportional improvements.

For the corresponding maximum displacement (Δlrup), the increase in thickness generally resulted in reduced deformation. The seamless configuration showed a 12% reduction, and the joint cover configuration decreased by 44.9%, indicating greater stiffness and limited elongation before failure. Conversely, the stitched and joint + stitched configurations showed modest increases of 30.3% and 8.4%, respectively, suggesting a slightly higher strain tolerance, possibly associated with localized damage progression rather than true ductility.

Overall, these relative differences confirm that increasing the number of layers significantly improves the mechanical efficiency and dimensional stability of the composite, particularly under compressive loading. The four-layer laminate not only resists higher loads but also exhibits smaller deformations, demonstrating superior structural integrity compared to the two-layer system.

An additional relevant parameter is the ratio between lateral and frontal displacement under the same load, which provides an indicator of the overall dimensional stability of the system. At the maximum level of 630 N, this ratio was approximately 0.91 for the two-layer composite, 0.73 for the four-layer composite, and 1.06 for the commercial material. These values reinforce the previous conclusions: the four-layer composite exhibits the best lateral confinement and the lowest tendency towards instability, while the commercial material showed lateral displacement values higher than the frontal displacement values, which may indicate eccentricity in load application or geometric imperfections that induce lateral bending. The two-layer composite, although exhibiting high deformability, maintains a lateral/frontal ratio of less than 1, demonstrating that the deformation, although high, is still dominated by axial compression.

From a micromechanical point of view, the results can be interpreted as follows: the two-layer composite, due to its smaller thickness and fewer reinforcing interfaces, has a more flexible structure and is subject to more pronounced transverse deformations; the four-layer composite, on the other hand, exhibits better stress distribution, less delamination, and greater structural confinement, ensuring greater stiffness and linearity; while the commercial material appears to have a more heterogeneous structure, with regions of higher and lower density, which explains the high initial lateral displacement and subsequent stiffening.

From a practical standpoint, these results indicate that the four-layer laminate is best suited for applications requiring high dimensional stability, low lateral deformation, and greater compressive strength, such as structural or mechanical support components. The two-layer composite, in turn, is more appropriate for situations where greater flexibility or energy absorption under load is desired, since its greater deformability can contribute to impact damping. The commercial material shows satisfactory performance, but its high lateral displacement and lateral/frontal ratio greater than 1 suggest that it may be sensitive to alignment imperfections or assembly variations during testing.

### 3.3. Correlation Between Tensile and Compression Behavior

The comparative assessment of tensile and compression results revealed a strong coherence between the material-level response of the laminates and the global structural performance of the helmet. In general, laminates that exhibited higher tensile stiffness and strength also presented reduced deformation under compressive loading, demonstrating a consistent mechanical relationship across different test scales.

The four-layer laminates, which showed the highest tensile stiffness and strength (greater Qrup and smaller Δlrup), correspondingly exhibited the lowest frontal and lateral displacements in the helmet compression tests. This direct relationship indicates that the mechanical integrity and interlaminar cohesion responsible for improved tensile stiffness also contribute to enhanced compressive rigidity and dimensional stability of the composite shell. The thicker laminates display greater fiber volume fraction, more efficient load transfer, and reduced susceptibility to local buckling or ovalization, resulting in a stiffer global structure that resists deformation under load.

In contrast, the two-layer laminates demonstrated lower tensile stiffness and higher elongation at failure, consistent with a more compliant behavior. This lower rigidity was reflected in the compression tests, where the two-layer helmet shells underwent larger displacements under equivalent loading conditions. The higher deformability observed in these thinner laminates suggests greater capacity for energy absorption but reduced structural integrity and stiffness, characteristics that may be advantageous for controlled impact dissipation but undesirable for quasi-static compressive stability.

This correlation establishes a positive relationship between tensile stiffness and compressive rigidity, as well as an inverse relationship between tensile ductility and helmet deformation. The consistent trends observed between tensile and compression results confirm the mechanical coherence of the system and validate the experimental methodology adopted. The findings demonstrate that the mechanical behavior determined from standardized tensile coupons ASTM D3039 [18] reliably predicts the overall structural response of the composite helmet under compression ABNT NBR 7471 [17], reinforcing the applicability of material-level data for structural design and optimization in natural-fiber-reinforced protective components.

### 3.4. Statistical Analysis and Interpretation

The statistical analysis presented in Table 5 shows significant differences between the conditions evaluated for the tensile and compression tests, confirming the impact of laminate thickness and joint configuration on the mechanical behavior of polyester matrix composites reinforced with jute fabric.

In tensile tests ASTM D3039 [18], the ANOVA test revealed highly significant main effects for both factors analyzed, number of layers (2 vs. 4) and type of configuration, as well as a statistically relevant interaction between these factors. The influence of the number of layers (*p* < 0.001) demonstrates that increasing the laminate thickness results in a significant increase in the maximum load supported (Qrup) and a proportional reduction in peak displacement (Δlrup), evidencing a more rigid and deformation-resistant material. The configuration factor also showed *p* < 0.001, indicating marked differences between the joining modes. The multiple comparisons test (Tukey HSD) showed that the seamless samples exhibited significantly higher resistance than the stitched samples, with differences far exceeding the minimum significant difference (HSD ≈ 0.10–0.12 kN). This drop in performance in the stitched samples is attributed to the discontinuity of the fibers caused by the holes for thread passage, which generate stress concentration and anticipate the onset of failure.

On the other hand, the joint + stitched configuration, which combines stitching with additional reinforcement by a bonded strip, showed intermediate behavior, reaching values close to those of the seamless laminate. This response indicates a structural synergy between the joining mechanisms: the stitching acts by restricting delamination, while the bonded strip redistributes stresses in the joint region, promoting greater interlaminar cohesion. The joint cover configuration, however, showed variable performance; in some conditions, it approached that of the seamless laminate, while in others it showed a slight reduction in strength, possibly due to stiffness discontinuities and shear concentrations at the adhesive interface.

In terms of deformation (Δlrup), the increase in the number of layers generally resulted in a reduction in overall deformability, reinforcing the gain in stiffness with thickness. However, the stitched samples showed greater peak displacement, which may be associated with a more gradual damage process, typical of laminates with localized progressive failure. Despite this, this increase in displacement should not be interpreted as a gain in effective ductility, since it occurs at the expense of structural integrity and load-bearing capacity.

The statistical significance of the interaction between thickness and configuration (*p* = 0.004) indicates that the benefit of additional lamination is not uniform for all joint geometries. In seamless and joint + stitched configurations, the strength gain when going from 2 to 4 layers is significant, while in stitched and joint cover configurations this effect is less pronounced or even non-existent. Overall, the ANOVA and Tukey HSD results confirm that the four-layer laminate exhibits significantly superior mechanical performance, especially when combined with joining techniques that preserve fiber continuity and interlaminar adhesion.

In frontal compression tests ABNT NBR 7471 [17], the effect of the number of layers was also highly significant (*p* < 0.001), with increased stiffness and reduced deformation as the laminate becomes thicker. Comparisons using Tukey’s test showed differences above the HSD (≈7.5 N/mm) between the two- and four-layer samples, confirming that the increased thickness confers greater dimensional stability and better load-bearing capacity. The “material” factor (composite × commercial helmet) showed *p* = 0.086, with no statistically significant difference at the 5% level. Even so, a trend towards greater stiffness is observed for the four-layer composite, demonstrating that the structure reinforced with natural fibers achieves performance comparable to that of commercial helmets, with the added benefit of being lighter and more environmentally sustainable.

Similar results were observed in the lateral compression test, where the effect of the number of layers was again significant (*p* < 0.001), with the minimum significant difference (HSD ≈ 8.4 N/mm) easily surpassed between the two- and four-layer samples. The increase in thickness resulted in significant gains in lateral stiffness and a reduction in deformation, reflecting greater interlaminar confinement and better distribution of transverse stresses. The material factor showed *p* = 0.072, not reaching statistical significance, although the trend of superiority of the four-layer composite remains. These results indicate that increasing the thickness of the laminate improves not only the axial strength but also the three-dimensional behavior of the system, reducing ovalization and lateral instability under compression.

### 3.5. Limitations of the Study

Despite the robustness of the experimental methodology and the consistency of the mechanical results obtained, some limitations of the present study should be acknowledged.

First, although the tensile behavior of the composite laminates was systematically evaluated according to ASTM D3039 [18], detailed microscopic fractographic analysis of the fracture surfaces—such as scanning electron microscopy (SEM)—was not performed. As a result, direct visualization of damage mechanisms including fiber breakage, matrix cracking, interfacial debonding, and the formation of resin-rich regions in stitched and bonded specimens could not be obtained. The interpretation of the tensile failure mechanisms discussed in this work is therefore based on consistent macroscopic fracture patterns observed after testing, combined with well-established micromechanical behavior reported in the literature for natural-fiber-reinforced polyester composites and composite joints.

Second, the higher stiffness and reduced deformation observed in the four-layer laminates were attributed to mechanisms commonly associated with increased laminate thickness, such as improved stress redistribution through the thickness and enhanced interlaminar constraint. While these mechanisms are well supported by previous experimental and numerical studies on synthetic and natural fiber composites, the absence of microstructural imaging techniques—such as optical microscopy or X-ray microtomography—limits the direct verification of void distribution, interlaminar defects, and local stress redistribution within the laminate. Future studies incorporating such techniques would provide a more detailed understanding of the internal structure and its relationship with the observed mechanical behavior.

Another important limitation concerns the long-term durability and biodegradation behavior of natural-fiber-reinforced composites. Although jute fibers are biodegradable by nature, their degradation rate within a thermoset polymer matrix is relatively slow and strongly dependent on environmental factors such as moisture ingress, ultraviolet radiation, temperature, and biological activity. In typical service conditions and under the standardized quasi-static mechanical tests employed in this study, the polymer matrix acts as a protective barrier, significantly delaying fiber degradation and preserving the mechanical integrity of the composite. Nevertheless, the present work focused exclusively on short-term mechanical performance and did not investigate long-term aging, environmental degradation, or biodegradation effects. Future investigations should therefore evaluate the evolution of mechanical properties of polyester/jute composites under accelerated aging, hygrothermal exposure, and biodegradation scenarios to quantify long-term strength retention and durability.

Finally, it should be emphasized that high-speed impact testing, which is essential for assessing energy absorption and safety performance in helmet applications, was not included in the experimental program. The present study was intentionally limited to quasi-static tensile and compression tests, following ASTM D3039 [18] and ABNT NBR 7471 [19], in order to evaluate material integrity, stiffness, and structural response under controlled loading conditions. While these tests provide valuable insights into the feasibility of natural-fiber composite helmet shells, dynamic impact tests at high strain rates are necessary to fully assess injury mitigation and real-world protective performance. Future work will therefore incorporate impact and fatigue testing to complement the quasi-static analysis presented herein.

## 4. Conclusions

This study investigated the mechanical feasibility of polyester composites reinforced with jute fabric for helmet applications, focusing on the influence of laminate thickness and joining strategy on tensile and quasi-static compression performance. Helmets manufactured by vacuum infusion using two-layer and four-layer laminate configurations were experimentally evaluated and compared with a commercial helmet material, following standardized tensile (ASTM D3039) and compression (ABNT NBR 7471) test procedures.

The tensile test results demonstrated that both laminate thickness and joining configuration significantly affect mechanical performance. Seamless specimens exhibited the highest tensile strength due to uninterrupted fiber continuity, while stitched-only specimens showed the lowest performance as a result of fiber disruption and stress concentration around sewing holes. Joint-cover specimens presented intermediate behavior, governed mainly by interfacial debonding and delamination in the overlap region. The hybrid joint (joint cover combined with stitching) exhibited a synergistic response, promoting more progressive failure modes and significantly higher rupture loads, particularly for the four-layer laminate. This behavior highlights the effectiveness of combining load-bridging mechanisms with through-thickness mechanical constraint to improve joint performance in natural-fiber composite structures.

Quasi-static compression tests revealed that laminate thickness plays a decisive role in the structural response of helmet shells. The four-layer composite consistently exhibited lower deformation and higher stiffness under both frontal and lateral loading when compared to the two-layer configuration, indicating improved load-bearing capacity and structural stability. The commercial helmet showed higher initial stiffness but a slight softening trend at higher loads, likely associated with manufacturing-induced pre-compaction and the onset of microstructural damage mechanisms such as matrix microcracking and local interfacial debonding.

In accordance with ABNT NBR 7471, compression tests were conducted up to a maximum applied load of 630 N, without inducing structural collapse. The maximum applied compressive load (Pmax = 0.63 kN) was therefore explicitly reported for all configurations, reinforcing the relevance of this parameter for helmet evaluation under normative quasi-static conditions. The results indicate that natural-fiber composite helmets manufactured by vacuum infusion can achieve mechanical responses comparable to those of commercial solutions under controlled compressive loading.

Overall, the findings demonstrate that polyester/jute composites produced by vacuum infusion present a promising potential for helmet shell applications, particularly when appropriate laminate thickness and hybrid joining strategies are employed. While the present work focused on quasi-static mechanical behavior, future investigations will extend this research by incorporating detailed microstructural characterization and high-speed impact testing to fully assess energy absorption, damage evolution, and safety performance under realistic impact scenarios. These future developments will further support the application of sustainable natural-fiber composites in protective equipment.

## Figures and Tables

**Table 1:** Geometrical dimensions of tensile test specimens according to ASTM D3039 [18]. Dimension E refers to the joint/overlap region length and does not correspond to the laminate thickness.

Dimensions (mm)	A	B	C	D	E	F
2 Layers	250	50	2.0	2.0	6.0	25
4 Layers	250	50	3.6	2.0	7.0	25

Note: The final laminate thickness was not defined by Dimension E. The average measured thickness was 2.0 ± 0.1 mm for two-layer laminates and 3.6 ± 0.2 mm for four-layer laminates, as obtained from vacuum-infused composite plates.

**Table 2:** Mean ± SD and coefficient of variation (CV %) for the tensile strength test of polyester/jute composites.

Configuration	Layers	Qrup (kN) ± SD	CV (%)	Elongation at Rupture, Δlrup (mm) ± SD	CV (%)
Seamless	2	0.80 ± 0.05	6.3	2.51 ± 0.23	9.2
Seamless	4	1.60 ± 0.07	4.4	2.21 ± 0.19	8.6
Stitched	2	0.12 ± 0.01	8.3	1.19 ± 0.09	7.6
Stitched	4	0.13 ± 0.01	7.7	1.55 ± 0.11	7.1
Joint cover	2	0.74 ± 0.03	4.0	1.56 ± 0.12	7.7
Joint cover	4	0.69 ± 0.04	5.8	0.86 ± 0.10	11.6
Joint + stitched	2	0.79 ± 0.04	5.1	1.66 ± 0.14	8.4
Joint + stitched	4	1.43 ± 0.08	5.6	1.80 ± 0.11	6.1

**Table 3:** Frontal compression test results ABNT NBR 7471 [19].

Load (N)	2 Layers (mm)	4 Layers (mm)	Commercial (mm)
30	1.11	0.66	1.0
130	4.14	2.33	2.06
230	6.7	3.77	4.43
330	9.08	5.22	6.16
430	11.18	6.74	7.77
530	13.16	8.29	9.13
630	15.09	9.97	10.76
**Pmax (kN)**	0.63	0.63	0.63

Note: Compression tests were conducted according to ABNT NBR 7471 [19], with load applied in incremental steps up to a maximum value of 630 N (0.63 kN). The specimens were not loaded until structural failure; therefore, Pmax corresponds to the maximum applied load defined by the standard.

**Table 4:** Lateral compression test results ABNT NBR 7471 [17].

Load (N)	2 Layers (mm)	4 Layers (mm)	Commercial (mm)
30	0.83	0.35	1.19
130	3.49	1.46	3.83
230	6.07	2.54	5.75
330	8.84	3.67	7.34
430	11.15	4.79	8.82
530	12.42	5.99	10.15
630	13.73	7.24	11.47
**Pmax (kN)**	0.63	0.63	0.63

Note: Compression tests were conducted according to ABNT NBR 7471, with load applied in incremental steps up to a maximum value of 630 N (0.63 kN). The specimens were not loaded until structural failure; therefore, Pmax corresponds to the maximum applied load defined by the standard.

**Table 5:** ANOVA and Tukey HSD results for tensile and compression tests.

Test	Factor	F (ANOVA)	*p*-Value	Tukey HSD Significant Differences (α = 0.05)	Least Significant Difference (HSD)
Tensile [18]	Layer (2 vs. 4)	68.7	<0.001	4 layers > 2 layers (for Qrup); 2 layers > 4 layers (for Δlrup)	≈0.12 kN (Qrup)
	Configuration	54.3	<0.001	Seamless > Stitched ≈ Joint Cover < Joint + Stitched	≈0.10 kN (Qrup)
	Interaction (Layer × Config.)	6.1	0.004	Significant for Seamless and Joint + Stitched (strength gains with thickness)	—
Compression—Frontal [19]	Layer (2 vs. 4)	41.5	<0.001	4 layers > 2 layers (higher stiffness, lower deformation)	≈7.5 N/mm
	Material (Composite vs. Commercial)	2.8	0.086	n.s. (no significant difference)	—
Compression—Lateral [19]	Layer (2 vs. 4)	58.9	<0.001	4 layers > 2 layers (higher stiffness, lower deformation)	≈8.4 N/mm
	Material (Composite vs. Commercial)	3.2	0.072	n.s.	—

## Data Availability

The original contributions presented in the study are included in the article; further inquiries can be directed to the corresponding author.
